# Key Considerations during the Transition from the Acute Phase of the COVID-19 Pandemic: A Narrative Review

**DOI:** 10.3390/vaccines11091502

**Published:** 2023-09-19

**Authors:** Piotr Rzymski, Maria Pokorska-Śpiewak, Teresa Jackowska, Ernest Kuchar, Aneta Nitsch-Osuch, Małgorzata Pawłowska, Mateusz Babicki, Jerzy Jaroszewicz, Leszek Szenborn, Jacek Wysocki, Robert Flisiak

**Affiliations:** 1 Department of Environmental Medicine, Poznan University of Medical Sciences, 60-806 Poznań, Poland; 2 Department of Children’s Infectious Diseases, Medical University of Warsaw, 02-091 Warsaw, Poland; maria.pokorska-spiewak@wum.edu.pl; 3 Department of Pediatrics, Centre for Postgraduate Medical Education, 01-813 Warsaw, Poland; tjackowska@gmail.com; 4 Department of Pediatrics with Clinical Assessment Unit, Medical University of Warsaw, 02-091 Warsaw, Poland; ernest.kuchar@gmail.com; 5 Department of Social Medicine and Public Health, Medical University of Warsaw, 02-007 Warsaw, Poland; aneta.nitsch-osuch@wum.edu.pl; 6 Department of Infectious Diseases and Hepatology, Faculty of Medicine, Collegium Medicum, Nicolaus Copernicus University, 85-067 Bydgoszcz, Poland; mpawlowska@cm.umk.pl; 7 Department of Family Medicine, Wroclaw Medical University, 51-141 Wroclaw, Poland; ma.babicki@gmail.com; 8 Department of Infectious Diseases and Hepatology, Medical University of Silesia, 41-902 Bytom, Poland; jerzy.jr@gmail.com; 9 Department of Pediatric Infectious Diseases, Wrocław Medical University, 50-367 Wroclaw, Poland; leszek.szenborn@umw.edu.pl; 10 Department of Preventive Medicine, Poznan University of Medical Sciences, 61-701 Poznań, Poland; jawysocki@pro.onet.pl; 11 Department of Infectious Diseases and Hepatology, Medical University of Białystok, 15-089 Bialystok, Poland; robert.flisiak1@gmail.com

**Keywords:** SARS-CoV-2, vaccination, antivirals, viral evolution, infectious diseases

## Abstract

The COVID-19 pandemic has been met with an unprecedented response from the scientific community, leading to the development, investigation, and authorization of vaccines and antivirals, ultimately reducing the impact of SARS-CoV-2 on global public health. However, SARS-CoV-2 is far from being eradicated, continues to evolve, and causes substantial health and economic burdens. In this narrative review, we posit essential points on SARS-CoV-2 and its responsible management during the transition from the acute phase of the COVID-19 pandemic. As discussed, despite Omicron (sub)variant(s) causing clinically milder infections, SARS-CoV-2 is far from being a negligible pathogen. It requires continued genomic surveillance, particularly if one considers that its future (sub)lineages do not necessarily have to be milder. Antivirals and vaccines remain the essential elements in COVID-19 management. However, the former could benefit from further development and improvements in dosing, while the seasonal administration of the latter requires simplification to increase interest and tackle vaccine hesitancy. It is also essential to ensure the accessibility of COVID-19 pharmaceuticals and vaccines in low-income countries and improve the understanding of their use in the context of the long-term goals of SARS-CoV-2 management. Regardless of location, the primary role of COVID-19 awareness and education must be played by healthcare workers, who directly communicate with patients and serve as role models for healthy behaviors.

## 1. Introduction

The coronavirus disease (COVID-19), caused by SARS-CoV-2 and first reported by the Chinese authorities in late 2019, rapidly became an emerging, evolving situation, spreading inevitably to other Asian countries and continents. The World Health Organization (WHO) first declared a Public Health Emergency of International Concern (PHEIC) on 30 January 2020 and considered COVID-19 as a pandemic on 11 March 2020 [1,2,3]. On 5 May 2023, it was announced that COVID-19 no longer had PHEIC status [4]. Within the three years, three months, and five days that passed in between, over 765 million SARS-CoV-2 infections were confirmed, with nearly 7 million deaths due to COVID-19 [5]. However, the true toll of the pandemic is likely a few-fold higher due to underdiagnosis and underreporting [6]. In addition, a range of symptoms can persist or emerge following acute SARS-CoV-2 infection, a condition known as post-COVID-19 syndrome, post-acute sequelae of SARS-CoV-2, or long-COVID [7], which also causes a considerable burden if one considers that its global prevalence has been estimated at 43% in the general population [8] and 25% in children and adolescents [9], although its accurate estimations are challenging due to both under- and overdiagnosis [10].

The COVID-19 crisis has led to the implementation of a hygiene regime, face masking, pursuing diagnostic testing daily, and imposing temporary school closures and national lockdown measures. Therefore, it has also had a broad societal impact, exacerbated pre-existing deep-rooted structural inequalities, caused numerous changes in different strata of life, and resulted in economic losses [11,12,13,14,15,16].

The emergence of SARS-CoV-2 has also led to an unprecedented scientific response encompassing essential research on diagnostic methods and studies of COVID-19 immunology, viral pathogenicity, and potential therapeutic targets [17,18]. Various pharmaceuticals (e.g., arbidol hydroxychloroquine, darunavir, lopinavir, favipiravir, remdesivir, ribavirin, ritonavir, interferons, dexamethasone, and tocilizumab) have been repurposed for COVID-19 treatment, with mixed effectiveness results [19,20,21,22]. The use of convalescent plasma was eventually abandoned due to the lack of clinical benefits observed in severely ill patients [23,24], while the effectiveness of different monoclonal antibodies has been dramatically impacted by SARS-CoV-2’s evolution [25,26]. The development and authorization of the first-generation anti-SARS-CoV-2 oral drugs (nirmatrelvir/ritonavir and molnupiravir) brought hope in 2022 that they may represent a turning point due to the possibility of their use outside the clinical setting [27]. However, the relatively high price of these pharmaceuticals and interactions with other drugs have been limiting factors in their use [27].

In 2020, great efforts also focused on developing vaccines to circumvent the need for social distancing and personal protective equipment [28]. This eventually led to their authorization in late 2020/early 2021 and the massive global vaccination campaigns pursued amid an influx of misinformation, fake news, and anti-vaccine propaganda [29]. It is estimated that COVID-19 vaccines prevented 19.8 million deaths in 2021 alone [30]. When the WHO announced that COVID-19 was no longer a PHEIC in May 2023, over 5.5 billion individuals had received at least one vaccine dose. Despite the high effectiveness of vaccines against severe disease and death [31,32], it soon became evident that due to a gradual decrease in serum antibodies, vaccination did not offer long-term protection from SARS-CoV-2 infection, leading to the recommendation of subsequent booster doses. In addition, viral evolution has led to the emergence of lineages, such as Omicron and its descendants, characterized by an increased ability to escape humoral responses [33,34,35]. Although the primary goal of COVID-19 vaccination is to decrease the rates of hospitalization, admission to intensive care units, and deaths [36] and is often achievable due to the extended duration of vaccine-induced cellular immunity and its lower susceptibility to viral mutations [37,38,39], some individuals may still experience severe COVID-19 due to a worse response to immunization because of age-related immunosenescence, primary or secondary immune deficiencies, and various lifestyle factors [40,41,42,43].

All in all, SARS-CoV-2 is far from being eradicated in the near future. It remains, as also emphasized by the WHO, a global health threat [4]. According to the official data, nearly 11 thousand COVID-19 deaths, with 64% in high-income countries, were reported in May 2023 since the WHO rescinded the PHEIC status. It is most likely that COVID-19 will become endemic, meaning that it will remain consistently present at predictable spread and occurrence rates. In this context, endemic does not necessarily imply that infection rates are low or that the disease is mild. For example, malaria is regarded as endemic in selected world regions, with its incidence rate and mortality remaining relatively stable since 2015, with approximately 600,000 deaths annually [44]. The endemic phase of COVID-19 will also require an appropriate management strategy and preparedness to decrease the disease burden systematically and by no means should it be equated with safe infections [45].

Therefore, in this narrative review, we highlight the essential issues regarding the benefits and future of COVID-19 vaccination, SARS-CoV-2’s evolution and its impact on the clinical significance of the disease, and the continuous need to pursue various control measures when exiting the acute phase of the COVID-19 pandemic but still existing with the virus, which can have profound effects on public health. The topics discussed here are pivotal to limiting the overall burden of SARS-CoV-2 and decreasing the effect that its infection may have on the most vulnerable risk groups during long-term co-existence with the viral pathogen. This discussion is particularly pivotal in guiding health policymakers and authorities when the COVID-19 threat is no longer heavily covered by the media and may be perceived by the general public as less important.

## 2. SARS-CoV-2 Is Here to Stay and Will Continue to Evolve

The priority of vaccinology has always been to decrease the clinical severity of infection. Preventing infection (whether symptomatic or asymptomatic) has been a secondary goal. The eradication of the pathogen is the most challenging task. As of today, smallpox remains the only human disease successfully eradicated due to vaccination campaigns [46]. SARS-CoV-2 will continue circulating in the human population, primarily because of the short-lived immune response following natural infection and vaccination and due to viral evolution. SARS-CoV-2 belongs to the RNA viruses, which exhibit higher rates of spontaneous mutation than DNA viruses [47]. The primary mechanism behind this phenomenon lies in the lower replication fidelity of the polymerase enzyme, ultimately leading to point mutations. Frequently, they do not affect virus biology or are deleterious to its further replication. A small minority of such mutations will provide fitness advantages and impact different aspects of virus biology, e.g., pathogenicity, infectivity, transmissibility, and antigenicity. The SARS-CoV-2 mutation rate has been estimated at 1 × 10^−6^–2 × 10^−6 ^ mutations per nucleotide per replication cycle [45,48], which is lower than the rates of various other RNA viruses, such as influenza viruses (3 × 10^−5^), human immunodeficiency virus (10^−4^ to 10^−5^), and hepatitis C virus (3.5 × 10^−5^ to 1.2 × 10^−4^) [49,50,51]. This is because SARS-CoV-2’s polymerase, similar to that of other coronaviruses, utilizes a proofreading 3′-to-5′ exoribonuclease of the nonstructural protein 14, a mechanism ensuring higher fidelity of replication, which is not present in the majority of RNA viruses. Nevertheless, the accumulation of point mutations in SARS-CoV-2 gives rise to novel lineages and sublineages that are competitive regarding transmissibility. An example of such a mutation is D614G in the spike protein, which emerged in late January/early February 2020 and increased SARS-CoV-2’s infectivity and soon became widespread [52].

The other process that can drive SARS-CoV-2’s adaptation is recombination. It results from the co-infection of the host cell with two genetically distinct viruses that, when recombined, produce viable hybrid progeny [53]. The odds of this process playing a more profound role in SARS-CoV-2’s evolution have increased over time due to the emergence and subsequent co-circulation of genetically divergent viral (sub)lineages, a phenomenon particularly evident in the Omicron era [45]. An example of such a SARS-CoV-2 recombinant that gained global relevance is the XBB that emerged from the recombination of the BA.2.10.1 and BA.2.75 sublineages [54]. The further accumulation of point mutations within this recombinant lineage gave rise to XBB.1.5, which became dominant in various world regions in 2023 [55].

**Figure 1:**
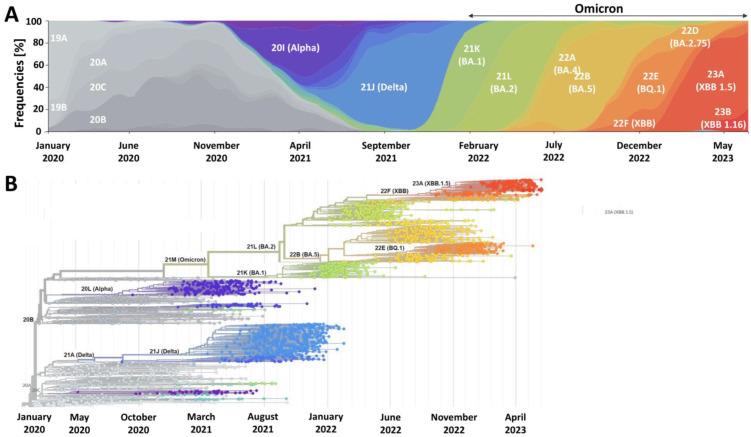
The emergence of SARS-CoV-2 variants over the course of the COVID-19 pandemic (**A**) and their phylogeny (**B**). The data and graphs were retrieved from Nextstrain.org [62].

SARS-CoV-2 will continue to evolve by accumulating advantageous point mutations and recombination events (Figure 1). It is pivotal to monitor its evolution and understand the key biological and clinical features of the emerging (sub)variants. One should note that the virus can become more adapted through various processes that depend on ecological and epidemiological factors. For example, mutations leading to the enhanced evasion of humoral immunity are more likely to be subject to higher selective pressure when the population immunity levels gradually increase. Such vaccine-bypassing and antibody-resistant mutations are observed in Omicron, and it can be expected that they might become a dominating pathway of SARS-CoV-2’s evolution when most of the world is either infected or vaccinated [56]. However, this does not imply the entire loss of the effectiveness of COVID-19 vaccines, since a vital role in the antiviral response is played by the vaccine-induced adaptive cellular immunity [57], which is less prone to evasion through viral mutations, as also evidenced in the case of various Omicron sublineages [58,59]. Nevertheless, it indicates that managing the SARS-CoV-2 burden will require the systematic administration of booster doses and consideration of updated variant-adapted doses, particularly if one aims to increase protection from symptomatic infection. In parallel, the implementation of novel vaccination strategies is needed to utilize other antigens than the spike protein due to a high number of mutations in its gene (second to the gene encoding nonstructural protein 3) [60]. In response to this need, approaches based on multiple antigen-targeted cell-mediated immunity have been suggested to overcome waning antibody responses and attenuate infectious breakthrough events and the disease severity of future SARS-CoV-2 variants [61].

## 3. Omicron Lineage Is Milder but Not Negligible

The SARS-CoV-2 variant belonging to the Omicron lineage was identified for the first time in November 2021 in Africa. It has been characterized by a large number of sense mutations, exceeding 30 in the gene encoding the spike protein, including 10 in the receptor-binding domain [63]. Its high transmissibility soon led to its global distribution, a rise in novel sublineages, and the replacement and ultimate extinction of previous viral variants (Figure 1). Numerous studies have consistently shown that the enhanced transmissibility of Omicron is due to its ability to better evade the humoral immunity of the vaccinated and individuals with a history of SARS-CoV-2 infection [64,65]. At the same time, there is mounting evidence that the Omicron lineage causes milder infections in humans. Firstly, experimental studies demonstrated its less efficient membrane fusion kinetics than previous SARS-CoV-2 lineages, preferential endocytic cell entry, and faster replication in the human bronchus, while less efficient in lung cells [66,67,68]. All of these features translate into decreased severity of infection. This has been clearly reflected in in vivo studies employing naïve animals, including rodents and non-human primates [69,70,71,72,73]. Epidemiological analyses of various human populations confirmed that Omicron infections are characterized by decreased lower respiratory tract involvement, reduced odds of hospitalization due to severe COVID-19, and less mortality [74,75,76,77,78]. Despite the emergence of subsequent Omicron sublineages, such as BA.4/BA.5 and XBB, the hospitalization and death risk remained lower compared to previous SARS-CoV-2 variants, such as Delta [79,80,81,82].

**Figure 2:**
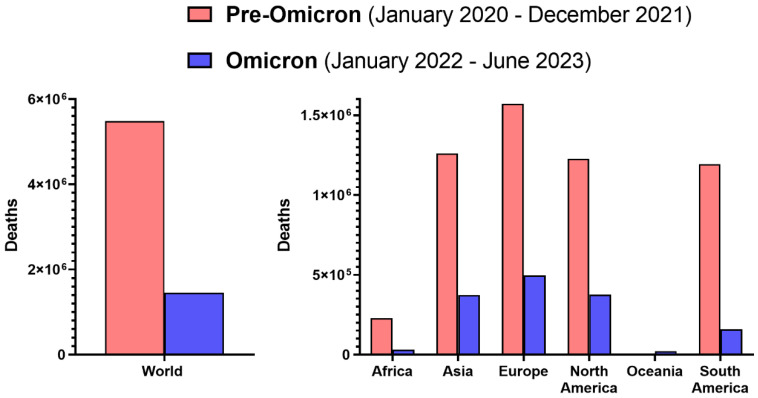
The global death toll of SARS-CoV-2 Omicron during the first 1.5 years of its dominance compared to the pre-Omicron period. Graphs were prepared based on data collected by Our World in Data [5].

Although Omicron causes statistically milder infections with a better clinical prognosis, contrary to some opinions, it should not be regarded as a negligible pathogen (Figure 2). Since the beginning of 2022, when Omicron became dominant in most world regions (Figure 1), until the first half of 2023, approximately 1.45 million deaths of COVID-19 patients were confirmed [5]. The mean monthly death rate in the pre-Omicron period amounted to 228 thousand, while, during the Omicron era, it fell over 2.5-fold to 86 thousand, which still was substantial and resulted in larger mortality than in the case of seasonal influenza, whose annual toll is estimated globally at 290,000–650,000 deaths [83]. This was also reflected by the results of a comparative study in patients requiring hospitalization, which demonstrated that Omicron infection is associated with an approximately 1.5-fold higher risk of in-hospital all-cause mortality than seasonal influenza viruses [84]. Moreover, due to high transmissibility and the ability to infect many individuals in a given period, the rising rates of hospitalization due to this variant in some countries were even higher than during the Delta wave [85]. Further, within the first 1.5 years of Omicron’s dominance, more than 478 million cases of infection were officially reported, representing 165% of cases in the first two years since early 2020 [5]. This indicates that Omicron, even if its infections are more frequently mild, can substantially affect absenteeism from work and school due to illness. Lastly, individuals infected with Omicron can also report, similarly to other SARS-CoV-2 variants, a range of persisting symptoms, collectively known as post-COVID-19 syndrome, post-acute sequelae of SARS-CoV-2, or long-COVID [7]. Although the odds of this condition with Omicron were reported to be significantly reduced compared to the Delta variant [86], its estimated rate in the post-acute cohort (6–15 months from infection) was 17%, most often characterized by chronic fatigue, but the range of symptoms encompassed systemic, cardiac, dermatologic, ocular, otologic, gastrointestinal, metabolic, musculoskeletal, neurologic, psychiatric and respiratory, and urinary [87].

Omicron is not a “natural solution” to the COVID-19 problem, as some have suggested [88], and should not be, along with various sublineages, regarded as a negligible pathogen. Despite a milder course of disease, it continues to cause substantial health and economic burdens, the management of which requires appropriate awareness, preparedness, and resources.

## 4. Future Viral Variants May Not Always Cause Milder Disease

It is challenging to predict the future clinical relevance of SARS-CoV-2. However, it is not certain that its further evolution will lead to a decrease in infection severity. As demonstrated by the recent study, the directions of change in intrinsic case severity across successive SARS-CoV-2 variant waves have been inconsistent. It has increased continuously from the early lineages, through the Alpha variant to the Delta lineage, to decrease substantially in the case of BA.1 Omicron and even further when BA.2 emerged [89]. This contradicts the notion that SARS-CoV-2’s transmissibility can only be enhanced at the expense of its pathogenicity, since the Delta variant, infection with which was characterized by increased severity, was significantly more transmissible than preceding lineages [90]. This advantage in spread was gained predominantly by higher viral loads [90,91]. In turn, the Omicron variant does not cause elevated viral loads in the respiratory tract compared to those observed for the Delta variant, while some studies have reported that these loads might even be lower [92,93,94]. In addition, it does not reveal a higher affinity to the angiotensin-converting enzyme 2 receptor and demonstrates attenuated fusogenicity due to decreased use of the cellular protease TMPRSS2, resulting in the greater utilization of the endocytic pathway during cell entry [67,95,96]. Its enhanced transmissibility is due to the efficient escape from humoral immunity in individuals with a history of SARS-CoV-2 infection and those who are vaccinated. SARS-CoV-2 may continue to evolve to develop greater escape from infection- and vaccination-acquired immunity. This could lead to its high transmissibility without a considerable increase in severity, particularly if the immune escape mostly concerns humoral and not cellular responses. However, considering that SARS-CoV-2 is most transmissible prior to symptom onset and at the beginning of the symptomatic phase [97,98,99], the mutation-enhancing viral loads could also lead to superior transmission yet be potentially accompanied by more severe infections due to a higher risk of hyperinflammation and disease severity under such a scenario [100].

Moreover, the viral evolution may lead to a gradual increase in fusogenicity, which is known to impact the disease severity [101]. This process has already been reported for more newly emerging Omicron subvariants such as BA.4/BA.5 and XBB, which demonstrate higher fusogenicity of the spike protein compared to early BA.1 and BA.2 SARS-CoV-2 [102,103,104]. Experimental studies have shown a close relationship between enhanced viral fusogenicity and pathogenicity [102,105]. Moreover, there is evidence that further Omicron subvariants, such as BA.5, reveal higher efficiency in infecting lungs than an early BA.1 subvariant [106]. Although epidemiological studies reveal some differences in clinical severity between the original and later Omicron subvariants, they consistently indicate that it remains reduced compared to the Delta lineage [107,108,109]. It is plausible that a history of immunization, be it SARS-CoV-2 infection, COVID-19 vaccination, or both, plays a protective role in attenuating the increased severity in the human population that would otherwise be expected.

When considering the future of SARS-CoV-2 evolution, one should note that it can also infect non-human hosts, including wild animals and livestock [110,111,112,113,114], and potentially return to the human population through contact with these species. In addition, Omicron can likely utilize a broader range of host species than other SARS-CoV-2 variants, while the risk of cross-species infection is higher due to increased human mobility than in the case of the pre-Omicron era, when various sanitary restrictions were imposed [115]. The clinical consequences of such retransmission to the human population are challenging to predict since mutation-driven adaptations to a new host may lead to decreased adaptation to the human environment but also to the better evasion of acquired immunity, including the cellular response, and thus higher susceptibility to severe disease [116,117,118,119].

In conclusion, predictions of the exact clinical trajectories of future SARS-CoV-2 (sub)variants should be made cautiously to avoid communication disregarding the relevance of this pathogen but also fear-promoting messages. SARS-CoV-2 requires continuous genomic surveillance conducted globally with data sharing in the open domain and accompanied by in vitro and in vivo studies on viral biology, pathogenicity, and the evasion of acquired immunity. This approach is essential for the timely implementation or modification of safety measures, including vaccines.

## 5. Vaccines Remain a Key Component of Primary COVID-19 Prevention

The benefits of COVID-19 vaccination are well documented. According to a mathematical modeling study, their administration prevented 19.8 million deaths in 2021 alone [30]. Numerous analyses encompassing a period preceding the dominance of the Omicron demonstrate the public health impact of COVID-19 vaccines in different world regions regarding averted deaths, hospitalizations, and infection [120,121,122,123,124,125,126,127,128]. According to a meta-analysis that included real-world studies conducted before Omicron’s emergence, the overall COVID-19 vaccine effectiveness against SARS-CoV-2 infection, COVID-19-related hospitalization, admission to the intensive care unit, and death was 89.1, 97.2, 97.4, and 99.0%, respectively, with better effectiveness against infection observed for mRNA vaccines [31]. Further, the majority of conducted studies have shown that vaccination reduces the risk of long-COVID [129,130,131,132,133,134,135,136].

However, vaccine effectiveness against infection decreased when the Omicron lineage emerged and became widespread due to its enhanced ability to escape humoral responses [32,137,138,139]. According to a meta-analysis, booster dose administration improved to some extent protection against symptomatic Omicron infection, reaching 57% within three months from administration but decreasing to 33% after six months [140]. However, COVID-19 vaccines remained highly effective in protecting against severe COVID-19 and death in the era of Omicron, and this effect was further demonstrated to be improved/restored by booster vaccinations [141,142]. As indicated in the meta-analysis, the real-world effectiveness of booster doses against severe disease caused by Omicron infection was 86% [140]. Another meta-analysis estimated the effectiveness of booster doses against Omicron infection and hospitalization at 70% and 89%, respectively, decreasing to 43% and 71% at 112 days or later [143]. In children and adolescent populations, the pooled effectiveness of two COVID-19 vaccine doses against symptomatic Omicron infection was 51 and 61%, respectively, with the pooled effectiveness against hospitalization of 70% [144]. As calculated in the UK, a booster dose program in autumn–winter 2021 averted 12.8 million cases, 1.1 million hospitalizations, and 290,000 deaths during the first three months of Omicron’s dominance in 2022 [145]. This clearly shows that although the authorized COVID-19 vaccines are still not optimal, they save lives, protect health, and decrease the economic losses caused by SARS-CoV-2.

In response to the emergence of the Omicron lineage, novel bivalent booster mRNA vaccines were developed and authorized in the second half of 2022. Their administration provided additional protection against symptomatic SARS-CoV-2 in immunocompetent persons who had previously received monovalent vaccines only [146]. Early estimates show that in adults aged 18–49 years, the effectiveness of a bivalent mRNA booster dose (with mRNA encoding primary spike protein antigen and BA.4/BA.5 spike protein) given 2–3 months earlier compared to no bivalent booster was 52% against symptomatic infection with the BA.5 Omicron subvariant and 48% against infection with XBB/XBB.1.5 [147]. A retrospective cohort study conducted in Israel confirmed that bivalent mRNA vaccines reduced hospitalization and mortality in individuals aged ≥65 years [148]. However, one should note that the effectiveness of these bivalent booster doses against infection with the Omicron variant was not as high as could be expected. This phenomenon may be due to immunological imprinting, according to which the immune system of those already vaccinated with monovalent vaccines was primed to respond to the ancestral strain of SARS-CoV-2. As a result, the administration of bivalent vaccines revoked the response to epitopes shared by Omicron (BA.4/BA.5) and the ancestral strain, rather than to unique epitopes of Omicron, as also directly demonstrated by the lack of BA.5-specific antibodies in the serum of individuals boosted with bivalent COVID-19 vaccines [149,150,151,152]. Therefore, future booster doses are likely to be monovalent and lack the index-virus antigen, also because they have been adapted to lineages currently considered extinct [153].

Importantly, studies conducted during Omicron’s dominance also show that vaccines continue to decrease the risk of long-COVID [154]. As estimated, the booster dose administration in autumn–winter 2021 resulted in a 68% reduction in newly diagnosed long-COVID cases in the first quarter of 2022, when Omicron was the dominant SARS-CoV-2 lineage [145].

In summary, the available evidence consistently demonstrates that all individuals should stay up to date with recommended COVID-19 vaccines, including receiving updated doses. COVID-19 vaccination reduces the overall burden of SARS-CoV-2 regardless of the dominating lineage, including the clinically milder Omicron. Nevertheless, it requires booster doses, including those based on updated antigens. Importantly, the mRNA platform enables the rapid manufacturing of novel versions of COVID-19 vaccines if such a need arises [155].

At the same time, there is a need to pursue efforts to develop vaccine candidates that could confer more durable protection against SARS-CoV-2 infection and be less prone to mutations in genes encoding the spike protein. One approach in this regard is focusing on the use of self-amplified mRNA vaccine candidates that aim to induce multiple antigen-targeted cell-mediated immunity in addition to neutralizing humoral responses in order to bypass waning antibody concentrations and attenuate the infectious breakthrough and disease severity of future SARS-CoV-2 variants [61]. Preclinical data show that using dual-antigen mRNA vaccines, encoding viral nucleocapsid and spike proteins, is superior in controlling SARS-CoV-2 (including the Omicron variant) in the lower and upper respiratory tracts than immunization with mRNA encoding exclusively the spike protein [156].

In addition, further efforts to develop effective intranasal COVID-19 vaccines are necessary as this route of administration may offer several advantages. Contrary to intramuscular vaccines, it can induce a mucosal immunity that plays a role in host defense in the upper respiratory airway, a primary entry site of viruses such as SARS-CoV-2, ultimately preventing virus infection, replication, shedding, transmission, and disease development and progression [157,158]. Secondly, the components of these vaccines can be absorbed through the mucosa, leading to systemic immunity [159,160]. Lastly, the intranasal route of administration is less invasive and painless and may translate into lower vaccine fears and improved acceptance [161,162]. Thus far, the development of safe and efficacious intranasal COVID-19 vaccines remains a challenge [163]. The intranasal COVID-19 adenoviral vaccine candidate failed to induce robust mucosal and systemic immunity in a phase 1 clinical trial [164], despite encouraging preclinical data [165]. In turn, the intranasal administration of lipid nanoparticles employed to encapsulate mRNA led to lung inflammatory responses and resulted in a high mortality rate [166]. These findings indicate the need for better preclinical models for mucosal immunity in humans and to develop strategies for the safe delivery of some vaccines, i.e., based on the mRNA platform.

## 6. Simplifying COVID-19 Booster Vaccination Will Improve Vaccine Acceptance and Intake

Given that COVID-19 vaccines will remain a primary strategy to decrease the health burden caused by SARS-CoV-2, it is essential to simplify the vaccination protocols, particularly regarding booster administration. Various observations demonstrate a significant decline in vaccination-induced antibodies within six months from the previous dose and indicate that biannual boosting with mRNA vaccines (most frequently used for this purpose) will induce the highest level of protection against infection [167,168,169,170]. Under such an approach, the risk of breakthrough infection over six years was estimated at 7–11%. In comparison, annual boosting would also substantially reduce the 6-year risk to 25–31%. In turn, delaying boosting beyond two years yielded cumulative risks of future infection nearly as high as foregoing boosting entirely [170].

However, one should bear in mind that interest in COVID-19 vaccines decreases with subsequent boosting doses. For example, by June 2023, 76% of the population had received at least one dose of the COVID-19 vaccine in the European Economic Area, the primary course of vaccination was completed by 73%, the first booster was received by 55%, the second one by 14%, while a third one only by 2% [171]. This trend has a multifactorial basis, including low perceived benefits of receiving a booster vaccine, a low subjective risk of severe COVID-19, disappointment in vaccines due to experiencing a breakthrough infection or adverse effects after the previous vaccine dose, and a loss of trust in health authorities during the pandemic [172,173,174,175]. All of these factors are more or less rooted in inappropriate communication on the role of COVID-19 vaccination in decreasing the overall SARS-CoV-2 burden on public and individual health as well as the economy. They may also arise from the various unknown factors regarding COVID-19 vaccines that existed when they were introduced (e.g., regarding the durability of immunity) and confusion about shifting public health guidelines regarding vaccine safety, changing the interval between doses, mixing particular vaccine brands, and introducing subsequent booster doses without knowing whether and when additional ones will be required [176]. These issues are currently clarified, allowing for the simplification of COVID-19 booster strategies, translating into lower vaccine hesitancy and better acceptance.

SARS-CoV-2 reveals a seasonal behavior, which is generally in line with that seen for other respiratory viruses, such as influenza viruses and respiratory syncytial virus [177,178,179]. For example, in Europe and the United States, the highest SARS-CoV-2 burden, i.e., infections, emergency visits, hospitalizations, and deaths, can be expected between autumn and early spring [179,180,181]. This strongly supports the notion that the administration of seasonal booster vaccines should be performed in a similar timeframe as in the case of influenza [179]. This timeframe also coincides with when RSV vaccines (currently gaining authorization for use in particular groups) will be recommended [182]. This creates an opportunity, being comfortable and less time-consuming from the perspective of those interested in vaccination, to offer seasonal booster COVID-19 vaccines simultaneously during the same visit as those against influenza and RSV. As recently shown, concurrently administering COVID-19 and influenza vaccines was not associated with additional safety risks and remained immunogenic, although marginally lower anti-S antibody levels were observed compared to booster COVID-19 vaccination alone [183]. Ultimately, the future may lie in combined vaccines. Some multicomponent mRNA vaccine candidates against COVID-19, influenza, and RSV have already entered clinical phases of testing (e.g., mRNA-1230; NCT05585632). The mRNA platform enables the development of updated COVID-19 boosters and seasonal influenza vaccines based on antigen selection approximately three months before an increased number of infections is expected [155]. This approach should increase the protection levels, not only against severe disease but also symptomatic infection, during a period when respiratory diseases are the most overwhelming for healthcare systems. A decision to select a novel version of the SARS-CoV-2 antigen should be made based on genomic surveillance data and the genetic divergence between currently and previously dominating viral sublineages.

Seasonal COVID-19 booster campaigns should preferentially target those at the highest risk of severe disease, including the elderly and individuals with comorbidities and immunodeficiencies, but also pregnant women and healthcare workers. This approach has also been recommended recently in a joint statement by the European Centre for Disease Prevention and Control and the European Medicines Agency [184]. We posit that these groups should be prioritized for reimbursed vaccines by local authorities. However, seasonal COVID-19 vaccines should also be made available for other eligible groups, e.g., through commercial distribution in a similar fashion to how influenza vaccines are offered in different world regions. Their administration also to those at lower risk of severe disease would decrease the risk of experiencing mild symptomatic infection and its consequences, such as long-COVID and being forced to abstain from work temporarily. It seems reasonable to recommend seasonal booster COVID-19 vaccinations, preferentially pursued at the same time as immunization against other respiratory viruses such as influenza and RSV.

## 7. Antivirals Represent a Strategy to Adapt to Long-Term Co-Existence with SARS-CoV-2

COVID-19 treatment depends on the severity of the infection and the presence of risk factors in infected patients. Pharmaceuticals targeting SARS-CoV-2 aim to inhibit viral replication and prevent disease progression to a more severe form [27]. For this purpose, they need to be applied in the early symptomatic phase [27]. However, ensuring that the targeted site is not subject to frequent mutations is critical. Otherwise, the effectiveness of such pharmaceuticals may soon decrease due to viral evolution. Such an effect has been observed in the case of various monoclonal antibodies either used to treat infection or as preexposure prophylaxis [185].

**Figure 3:**
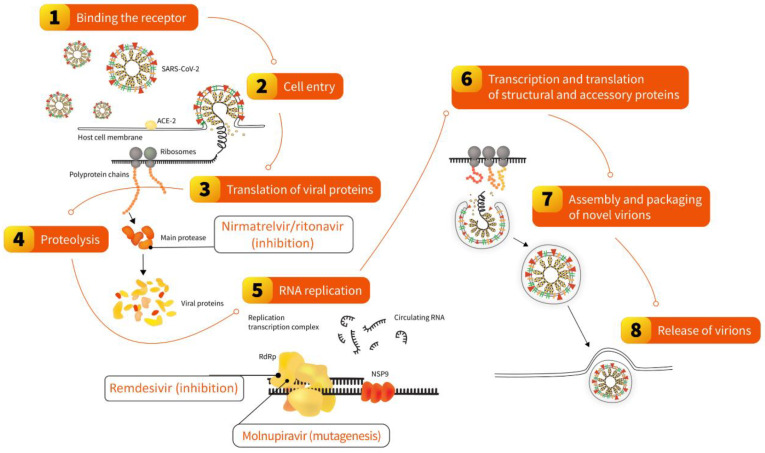
Steps of the SARS-CoV-2 replication cycle in the human cell disrupted by oral antivirals nirmatrelvir/ritonavir and molnupiravir and intravenously administrated remdesivir. The scheme was used and modified with permission [27].

In 2022, two oral antivirals specifically targeting the SARS-CoV-2 replication cycle were recommended for use in different world regions: nirmatrelvir/ritonavir and molnupiravir [27] (Figure 3). Both require 5-day treatment initiated no later than five days from symptom onset. The former inhibits the main SARS-CoV-2 protease, pivotal to processing polyprotein precursors, ultimately leading to the inability of the virus to replicate. This mechanism is ensured by nirmatrelvir, which is extended in the presence of a low dose of ritonavir, acting as an inhibitor of CYP3A-mediated metabolism. The pivotal phase 2–3 double-blind, randomized, controlled trial on nirmatrelvir/ritonavir, conducted in symptomatic, unvaccinated, nonhospitalized adults at high risk for progression to severe COVID-19, reported a reduction in hospital admission or death by 97% relative to a placebo. Experimental studies demonstrated that it remains effective against various Omicron subvariants [185], observations that were further confirmed by clinical trials and real-world studies conducted in different populations and reporting reduced hospitalization and mortality in treated patients [186,187,188]. In addition, a large cohort study found that treatment with nirmatrelvir/ritonavir during the period dominated by the Omicron variant was associated with a reduced risk of long-COVID regardless of vaccination status and prior infection [189].

Molnupiravir is a small-molecule ribonucleoside pro-drug of N-hydroxycytidine [190], which was tested prior to the COVID-19 pandemic for potential use against SARS-CoV-1 and MERS-CoV [191,192]. Its mechanism of action is based on so-called lethal mutagenesis, the process in which viral RNA-dependent RNA polymerase is misdirected to induce transition mutations throughout the genome during viral replication, ultimately leading to errors deleterious for the virus. A double-blind, randomized, placebo-controlled phase 3 clinical trial in symptomatic, unvaccinated, nonhospitalized adults showed that the risk of death was 89% lower in the group receiving molnupiravir for five days [193]. However, no clinical benefit was found in the clinical trial involving hospitalized patients [194]. These studies were conducted during the dominance of viral variants other than Omicron. Nevertheless, experimental in vitro studies demonstrated that molnupiravir remains efficacious against this variant [185], further confirmed in clinical trials and real-world studies also involving hospitalized COVID-19 patients [186,195,196]. Moreover, molnupiravir use was also associated with a reduced risk of long-COVID regardless of vaccination status and history of previous SARS-CoV-2 infection [197].

Apart from oral antivirals, which can be used in outpatient and inpatient settings, an important treatment option in patients hospitalized with COVID-19 includes remdesivir, an intravenously administrated non-canonical nucleotide developed prior to the COVID-19 pandemic (Figure 3). It acts as an inhibitor of the RNA-dependent RNA polymerase of RNA viruses of several families, including Paramyxoviridae, Filoviridae, and Coronaviridae. Based on evidence from clinical studies, remdesivir was authorized in 2020 by various health authorities to treat COVID-19 in adults and adolescents (>12 years with weight ≥ 40 kg) who require oxygen therapy. It can also be used in adults who do not require oxygen supplementation but represent a high-risk group for severe COVID-19 [198]. Experimental in vitro studies have shown that it remains efficacious against Omicron’s sublineages, including BQ.1.1 and XBB [185]. This was also confirmed in the real-world analysis in which remdesivir use in patients hospitalized during Omicron’s dominance was an independent predictor of lower mortality, similar to the period dominated by the Delta lineage [199].

In summary, antivirals such as nirmatrelvir/ritonavir, molnupiravir, and remdesivir retain their effectiveness against the novel SARS-CoV-2 sublineages and continue to be important elements of COVID-19 therapy. It is pivotal to ensure their availability, particularly when an increased number of SARS-CoV-2 infections can be expected (e.g., during the autumn–winter season in the temperate zone). In this regard, oral antivirals are the pharmaceuticals of choice as they reduce healthcare costs through decreased hospitalization rates [200,201,202]. At the same time, it is important to pursue research on the potential benefits arising from therapies based on the combination of antivirals, assessed mostly as case reports [203] or in vivo rodent studies [204]. Such combinations may slow the emergence of resistance mutations, as already evidenced for other pathogens [27,205,206]. This may be particularly of interest in the case of immunocompromised patients since they are often characterized by an extended time of viral elimination, even when treated with available antivirals [203,207,208,209]. The high-dosing regimen recommended for nirmatrelvir/ritonavir (three tablets administrated twice a day for five days) and molnupiravir (four capsules every 12 h for five days) also represents a challenge due to the risk of missing a dose or inappropriate adherence, which is not possible to directly control outside the clinical setting. Moreover, nirmatrelvir/ritonavir tablets and molnupiravir capsules are relatively large (8.5 × 17.5 mm and 7.6 × 21.8 mm, respectively [210,211]) and cannot be chewed or crushed and may be difficult to swallow by selected patients, including the elderly, for whom this issue has been particularly recognized [212,213]. Improved formulations, requiring reduced dosing and based on a smaller size of swallowed tablets/capsules, would be ultimately desired. Simultaneously, continued efforts to increase the portfolio of anti-SARS-CoV-2 pharmaceuticals, preferentially administrated orally and acting on different viral targets, are highly encouraged [27].

## 8. Leaving No Country Behind: Low-Income Regions Require Better Access to COVID-19 Vaccines and Antivirals

Considering that COVID-19 vaccines and SARS-CoV-2 antivirals remain essential tools during the transition from the acute phase of the pandemic, it is pivotal to pursue efforts to increase their availability and willingness to use in low-income countries. Although the case–fatality ratio for various low-income areas, e.g., the African continent, remains below that observed globally [214], this does not imply that SARS-CoV-2 is a negligible pathogen. This is also because the reasons behind such epidemiological phenomena are unclear, with various hypotheses put forward, including cross-protection from other infections or younger populations than in other world regions [215,216,217]. However, low-income countries likely have the highest rates of underreported COVID-19 cases (including severely diseased) and COVID-19 mortality [218,219]. At the same time, they are represented by low vaccination rates, with approximately 25% of the population of low-income countries having completed an initial vaccination protocol [5]. This is due to several factors, including insufficient supply, limited local vaccine production, inequitable distribution, weak healthcare systems, a low perceived risk, and high vaccine hesitancy [220].

**Figure 4:**
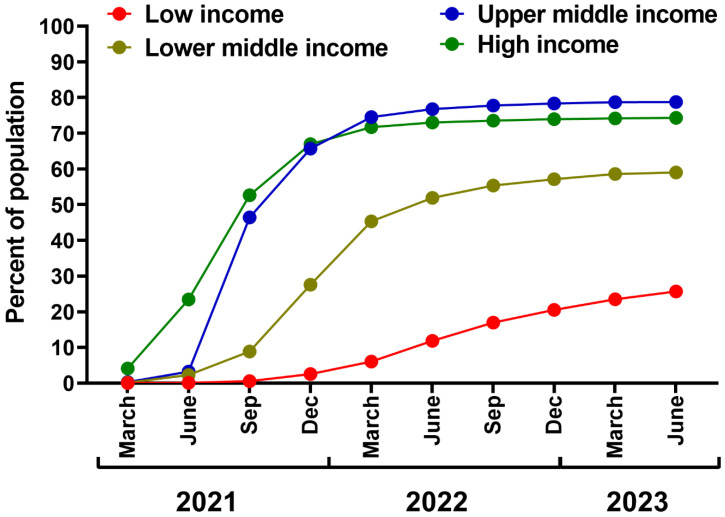
The percentage of the population with completed initial COVID-19 vaccination protocol by economic group. Prepared based on data retrieved from Our World in Data [5].

The efforts should be continued to improve vaccine equity in low-income countries through better support of humanitarian initiatives from high-income countries, such as the COVID-19 Vaccines Global Access (COVAX) initiative. As of early 2023, it has delivered 1.88 billion doses [221], despite its initial target to deliver 2 billion doses in 2021 [222]. This results in great discrepancies in vaccination rates in the world, i.e., 2.5 years after COVID-19 vaccine authorization (June 2023), the percentage of the population with a completed initial vaccination protocol in low-income countries was similar to that already reached by high-income regions after six months of the campaign in 2021 (Figure 4). This failure resulted mainly from the subdued efforts of wealthy regions, vaccine nationalism, and trade between high-income countries [36,223,224], summarized by the WHO’s Director-General as a “handful of rich countries gobbling up the anticipated supply as manufacturers sell to the highest bidder, while the rest of the world scrambles for the scraps” [225]. Notably, the most considerable benefits of COVID-19 vaccination, in terms of averted deaths, have been demonstrated for high-income and upper–middle-income regions, likely due to better logistics, swift rollout, and improved access to highly efficient mRNA vaccines [30]. These findings also underline the need for vaccine aid and support in regions of lower income (Figure 4). As estimated, universal vaccination in low-income and lower–middle-income countries with three doses of a mRNA vaccine would prevent as many as 1.5 million COVID-19-related deaths in a period already dominated by the Omicron lineage [226].

One should also note that prior to Omicron’s emergence, researchers continuously warned that vaccine inequity during the COVID-19 pandemic not only reflected a moral crisis but also increased the odds of the emergence of novel, problematic SARS-CoV-2 variants [36]. Although the exact origins of the Omicron lineage remain unknown, it is suggested that it may arise during the infection of immunocompromised individuals (e.g., HIV/AIDS patients) or even cross-infection between a group of them due to extended viral replication and the selection of neutralization resistance mutations in such subjects [227,228]. In addition, a study conducted before the emergence of Omicron has shown that the mutation frequency positively correlates with the percentage of unvaccinated individuals in a population, with the highest frequency found for regions with vaccination rates below 10–20%. In turn, the rate of individuals who completed a primary vaccination course in Africa, which has the highest population of people living with HIV (predominantly in the Sub-Saharan area), was approximately 5% at the time of Omicron’s identification (compared to nearly 55% in the USA, 65% in the European Union, and 50% in Oceania). Although infections with Omicron are milder compared to the SARS-CoV-2 lineages preceding it, a lesson must be learned, particularly if one considers that viral genomic surveillance in low-income countries is limited [229]. When various health authorities issue novel recommendations regarding COVID-19 vaccination [153], there is no reason to shape them differently for low- and high-income regions, since COVID-19 remains a global issue and should be treated equally regardless of one’s origin or ethnicity [224]. Importantly, it is crucial to ensure that low-income regions continue to move away from aid dependence through various mechanisms enabling the local production of vaccines, including those based on innovative technologies such as mRNA platforms. This could be done by building on the existing capacity, developing sustainable financing mechanisms and quality control systems, prioritizing research funding and regional integration, and collaboration conceptions based on technology co-creation and co-ownership, as discussed elsewhere [155]. The improvement of the manufacturing and supply of vaccines based on technologies such as mRNA would also be necessary outside the COVID-19 realm if one considers their potential to deliver preventive tools against other infectious diseases [155,230], some of which are particularly burdensome in low-income countries and have zoonotic origins [231,232].

Simultaneously to vaccine equity, it is essential to improve accessibility to SARS-CoV-2 antivirals in low-income countries, particularly those available in the oral form [233]. The first-generation oral antivirals are relatively expensive [234], highlighting the need for aid in delivering these pharmaceuticals to low-income regions, developing generic versions of these drugs, and pursuing efforts to produce them locally [27]. This is particularly important since these antivirals can substantially reduce the risk that infected patients will require specialized healthcare, access to which is limited under low-income resources.

All of these efforts require integration with improved education and awareness campaigns to fight vaccine hesitancy, educate on infectious diseases, including COVID-19, and build trust in local authorities and vaccine manufacturers. These goals will also likely require external support, bringing together experience from vaccination campaigns in developed regions and local specificity. The improvement of accessibility to pharmaceuticals and their acceptance in low-income areas should be an integral part of a strategy for pathogen management in high-income countries if one considers that, in an increasingly connected modern world, the risks arising from infectious disease can be globally shared [235].

## 9. Healthcare Workers Play a Crucial Role in Maintaining Public COVID-19 Awareness

Healthcare workers are a pivotal part of health communication as they interact directly with patients, including those at high risk of various diseases such as severe COVID-19. Moreover, they serve as role models of healthy behaviors, including vaccination decisions [236]. In fact, their role in general COVID-19 awareness may even be more influential during the transition from the acute phase of the pandemic. This is because, earlier, regular communication with patients in this regard was likely curbed due to the allocation of healthcare resources to fight COVID-19, social distancing, closures of primary care units, and increased stress experienced by healthcare workers [237,238,239,240]. At the same time, COVID-19 received high media coverage, with various information on preventive measures often reported daily [241]. Vaccination campaigns, the lifting of sanitary restrictions, the spread of the clinically milder Omicron lineage, and the emergence of other issues of public importance (e.g., a war in Ukraine) translated into a decreased interest in COVID-19 in traditional and social media. This may have led to the false assumption that COVID-19 is no longer a threat requiring any preventive measures, e.g., seasonal vaccinations.

Therefore, it is pivotal to ensure that healthcare workers continue their efforts to communicate the risks for particular groups of patients, follow the recommendations on vaccination, and communicate them further in an understandable manner. As recently noted in the joint statement by the European Centre for Disease Prevention and Control and the European Medicines Agency, seasonal COVID-19 vaccination of healthcare workers should be considered because they have a higher risk of exposure to SARS-CoV-2 while playing a key role in the functioning of the healthcare system [184]. However, their decision to vaccinate is also likely to be influential for their patients [242]. Therefore, ensuring an appropriate education level on COVID-19 vaccination among healthcare workers, including primary physicians, is crucial. As shown in a study led by the WHO/Europe, healthcare workers are more confident in recommending COVID-19 vaccines to their patients if they undergo dedicated online training on how to communicate with patients regarding the vaccination [243]. They will also likely be more confident in discussing COVID-19’s risks with particular groups of patients after completing the training course, updating them on current SARS-CoV-2 sublineages in circulation and their clinical relevance. Moreover, it is essential to continue efforts to raise awareness of the effectiveness of non-pharmacological measures, such as face masks and hand hygiene, in limiting the spread of SARS-CoV-2, as well as other respiratory infections, particularly when feeling unwell or when in contact with elderly or immunosuppressed patients during periods of increased transmission of SARS-CoV-2.

One should note that apart from physicians, an increasingly important role in vaccination is played by pharmacists [244,245,246]. A study conducted in the US demonstrated that one in four people who refused to receive influenza and pneumococcal vaccines could eventually decide to receive a vaccination after consultation with a pharmacist in a pharmacy [247]. As calculated in other analyses, including pharmacists in consultation services among seniors for influenza vaccination is cost-effective and improves vaccination rates in this group [248]. Therefore, political and organizational barriers should not limit pharmacists’ participation in COVID-19 vaccination campaigns. Therefore, the local authorities should consider increasing the rights of pharmacists in qualifying, prescribing, and vaccinating patients against COVID-19, ultimately simplifying seasonal vaccination campaigns and likely translating into higher vaccination rates.

## 10. Conclusions

The acute phase of the COVID-19 pandemic may be over, as reflected by the WHO’s decision to rescind its PHEIC status in May 2023, but SARS-CoV-2 continues to spread, evolve, and cause economic and health burdens. There are still many uncertainties regarding the future of COVID-19, its severity, and its global impact in the long-term perspective. Therefore, as highlighted in the present paper, it requires sustained genomic surveillance and the promotion of prevention strategies that are as simplified as possible, continuously supported by the healthcare community, and accessible also in low-income regions. All of these elements should be a part of the strategy to adapt to long-term co-existence with SARS-CoV-2 in a manner that prevents healthcare systems from being overwhelmed.

## Data Availability

Not applicable.
